# Guidable Thermophoretic Janus Micromotors Containing Gold Nanocolorifiers for Infrared Laser Assisted Tissue Welding

**DOI:** 10.1002/advs.201600206

**Published:** 2016-09-01

**Authors:** Wenping He, Johannes Frueh, Narisu Hu, Liping Liu, Meiyu Gai, Qiang He

**Affiliations:** ^1^ Key Laboratory of Microsystems and Microstructures ManufacturingMinistry of EducationMicro/Nano Technology Research CentreHarbin Institute of TechnologyYikuang Street 2Harbin150080P. R. China; ^2^ Mental Health Centre1st Affiliated Hospital of Harbin Medical UniversityHarbin150001P. R. China; ^3^ Queen Mary University of LondonSchool of Engineering and Materials ScienceMile End, Eng, 215LondonE1 4NSUK

**Keywords:** diffusion based temperature measurement, Janus capsule, laser tissue welding, polyelectrolyte multilayer, thermophoretics

## Abstract

Current wound sealing systems such as nanoparticle‐based gluing of tissues allow almost immediate wound sealing. The assistance of a laser beam allows the wound sealing with higher controllability due to the collagen fiber melting which is defined by loss of tertiary protein structure and restoration upon cooling. Usually one employs dyes to paint onto the wound, if water absorption bands are absent. In case of strong bleeding or internal wounds such applications are not feasible due to low welding depth in case of water absorption bands, dyes washing off, or the dyes becoming diluted within the wound. One possible solution of these drawbacks is to use autonomously movable particles composing of biocompatible gold and magnetite nanoparticles and biocompatible polyelectrolyte complexes. In this paper a proof of principle study is presented on the utilization of thermophoretic Janus particles and capsules employed as dyes for infrared laser‐assisted tissue welding. This approach proves to be efficient in sealing the wound on the mouse in vivo. The temperature measurement of single particle level proves successful photothermal heating, while the mechanical characterizations of welded liver, skin, and meat confirm mechanical restoration of the welded biological samples.

## 1 Introduction

Wounds are introduced to the human body or animals by injury or surgery. Smaller wounds can self‐heal over time without any treatment, while larger wounds require treatments to recover. The more time it takes to close and recover the wounded tissues, the more likely the body will get infected by bacteria introduced from the surrounding environment.^1^, ^2^ Therefore, an efficient means to assist wound sealing is of great importance not only in accelerating the sealing process, but also in minimizing the chance of contamination.^1^, ^2^ Wound sealing is until now performed by stitching for larger wounds or using band plasters for smaller ones.^3^ The stitching approach has severe drawbacks since it mechanically forces the tissue together causing kinks in the healed tissues, and in addition it creates new wounds and trauma.^4^ Novel approaches such as nanoparticle‐based wound sealing are promising, but since the gluing happens immediately, no further correction is possible when the wound is closed by mechanical pressure at the first time.^4^, ^5^

Laser based tissue welding on the contrary offers a defined wound sealing only when the laser is applied.^6^ In addition, it disinfects the wound, due to high local temperatures.^7^ Laser tissue welding has been under investigation since the end of the 1970s.^3^ The system exploits collagen denaturation, which happens between 50 and 70 °C.^3^, ^8^ The denaturation allows for a tissue softening and melting, whereby a temperature decrease allows for condensation and a hard wound closure.^3^, ^6^ Various approaches such as utilization of water absorption bands in UV and IR areas were used along with organic dyes but also gold nanorods or nanoparticles were used as near infrared adsorption dyes.^7^, ^8^, ^9^ The use of solid gold nanoshell dyes containing a dielectric material in the middle to tune infrared adsorption was also performed recently.^6^

The aforementioned approaches follow the idea to simply add a dye to the wound. Such an approach falls short when the wound is strongly bleeding, and therefore the dye would be washed off before it can be heated significantly to create a seal. The use of autonomously movable micro‐ and nanomotors offers a solution for such problems, since these can move with speeds up to hundreds of micrometers per second.^10^, ^11^ In addition they offer magnetic guidance allowing for controlled motion even upstream of bleeding sites.^12^

**Scheme 1:**
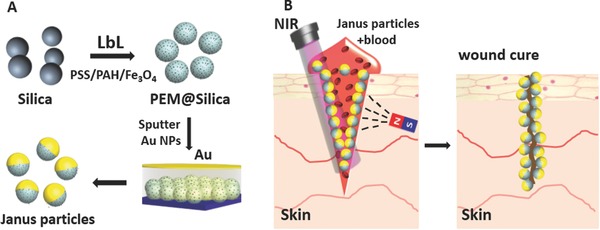
A) Production of Janus composite particles by LbL self‐assembly of PEM and magnetite nanoparticles followed by sputter coating with gold and resuspension in water. B) Laser tissue welding with magnetic assistance, due to magnetite particles being homogeneously distributed in the particles the particle orientation is random during welding.

We present hereby a Janus type of tissue welding dye, which offers dual functionality. The utilized particles comprised of polyelectrolyte multilayers,^13^, ^14^ and superparamagnetic iron oxide nanoparticles,^15^ which were acquired by coating silica particles. With subsequent dissolution of the core capsules can be obtained which offer similar photophysical properties.^16^ Prior to dissolving the core, the coated particles were adsorbed on a glass slide, whereby one side was sputtered with gold. The particles and capsules are able to move in solution due to thermophoretic effects. These particles and capsules can be guided by rare earth magnets within blood and tissue fluids of damaged and cut meat samples, and the meat can be fused using near infrared laser. The utilized gold and magnetic nanoparticles are biocompatible, as they are already used as magnetic resonance imaging contrast agents, while the used polyelectrolytes are harmless in complexes (PEM is such a complex).^17^, ^18^, ^19^ The structural buildup of the Janus particles and capsules is displayed in **Scheme** **1**. In addition, we prove that a successful wound sealing can be performed utilizing these particles in animal experiments.

## 2 Results and Discussion

### 2.1 Characterization

**Figure 1:**
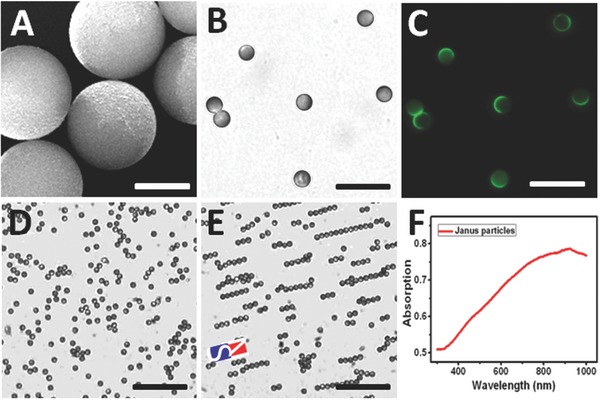
A) SEM image of PEM–magnetite–gold Janus composite particles, which contain superparamagnetic Fe_3_O_4_ nanoparticles (scale bar = 3 μm), confocal optical images of Janus capsules B) bright field, and C) FITC channel (FITC labeled PAH was used) (scale bar = 25 μm); D) free swimming Janus particles and E) guidance of Janus particles by magnetic fields; F) UV–vis‐NIR absorption spectra of Janus capsules.

Electron and confocal images prove the Janus structure (**Figure** **1**A–C). Applying a magnetic field close to the sample allowed for Janus particle orientation as well as steering (Figure 1D,E). UV–vis‐NIR absorption spectra show a peak in the NIR region close to the utilized laser wavelength (Figure 1F). The Janus structure is in agreement with earlier studies of photothermal effects on Janus particles.^20^ Due to the strong NIR absorption thermophoretic motion away from the laser as was found in Video S1 of the Supporting Information. Apart from the thermophoretic motion, the thermo convection generated by optical vortex effects can shoot particles out of the laser focus, such effects require however a 2× higher laser intensity as shown in Video S2 of the Supporting Information.

As Figure S1A of the Supporting Information shows photothermal heating determined by particle diffusion using a focused TEM00 mode laser shows mainly a decrease in particle diffusion, until the particles are ejected from the focus for both, pristine and Janus particles. The only way to determine the particle temperature reliably is to normalize the particle diffusion of pristine and Janus particles, whereby the pristine particles were regarded as not heating. In addition, it is necessary to determine the laser power necessary for starting optical trapping and to take its values into account for the normalization. In the observed case the optical trapping of the Janus particles started at 17.5 mW, while one of the pristine particles started at 22 mW, for this reason the diffusion of Janus particles was normalized with the diffusion (*D*) of pristine particles of a 4.5 mW higher laser power.

Such an approach is necessary since the gold nanoparticles interact with light much stronger compared to silicon dioxide and therefore the achieved temperature as well as trapping strengths are higher. Neglecting such effects can cause physically not meaningful results like determined temperatures being below the freezing point of water or first an increase, then a decrease in detected temperature, as Figure S1B of the Supporting Information shows. Since multimode lasers showed a decrease in *D* but no corner frequencies, the results obtained from these lasers are not regarded interpretable in this study.

**Figure 2:**
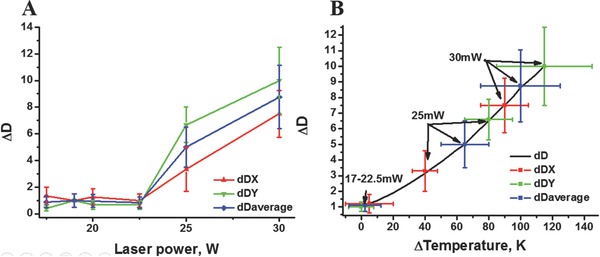
Temperature determination of Janus particles versus laser power. A) Change in diffusion values of 5 μm Janus particles normalized with the trapping corrected diffusion of pristine 5 μm particles versus laser power. B) Change in temperature (Δ*T*) versus increase in diffusion (Δ*D*). Despite initial Janus particle based temperature splitting between X and Y a rotation at higher laser powers causes the 2 values to come together again.

Normalized particle diffusions proved an increase of particle temperature starting from 22.5 mW. The temperature was found to increase steeply until at 30 mW the diffusion rate surpasses the boiling point of water. It is noted, that the total temperature is ≈135 °C but due to the absence of clear nucleation points, as well as the high Poisson pressure necessary to form a small bubble no boiling was observed. The finding that Janus particles offer (when they are in configuration like Figure 1B) uneven diffusion in X and Y dimension which is in line with the observation reported by He et al.^21^ This finding seems to contradict reports^22^ of others reporting a shift of the particle in a way that the gold covered side of the Janus particles faces the laser. In such a system one should not be able to detect diffusion differences between X and Y dimensions. The solution of this contradiction is that our particles are heavier than the ones reported in reference^22^ and therefore rotate due to surface friction at higher laser powers. This is indicated at 30 mW in **Figure** **2**B where the X and Y dimensions come into closer proximity than at lower laser powers, despite one expects the values to increase at higher laser powers. The temporary differences in detected X and Y temperatures are explained by the differences in local gold layer absorption which is due to the evaporation conditions thicker in Y than in X direction.

It is worth to point out that the observed laser based heating presented here is in line with the absorption and heating rates of nanoparticles, whose heating rate was observed not by whole particle motion, but rotational changes, as well as with simulated or lipid bilayer disordering studies.^23^, ^24^, ^25^, ^26^, ^27^ Also temperature measurements of Janus particle based cancer treatment agents resulted in similar values like the ones presented here.^21^ The authors would like to point out that the use of a 3D alignment stage allowed detecting a larger laser power range compared to 2D alignment stages used by He et al,^21^ which only investigated single measurement points.

Investigating not only single particles but a large number of particles with a IR camera on a dry glass slide whereby the particles were heated with a 4 × objective, one observes a steady increase in temperature which reaches >90 °C at ≈230 mW laser power as shown inFigure S2 of the Supporting Information. In water and on meat the values are significantly lower due to water based heating and IR shielding. Figure S3 of the Supporting Information proves the highly local heating of the samples due to laser assisted heating.

The achieved temperature rise is high enough to thermally denaturate collagen and albumin, since collagen starts to denaturate at ≈70 °C.^3^, ^28^ The causes for differences in detected temperatures between Figure 2 and Figures S1 and S2 of the Supporting Information are due to the fact that the laser point in Figure S1 of the Supporting Information is smaller than the detection area. In addition, one integrates over a large area which is not completely covered with particles but contains lots of air, water, and/or meat between the particles.

**Figure 3:**
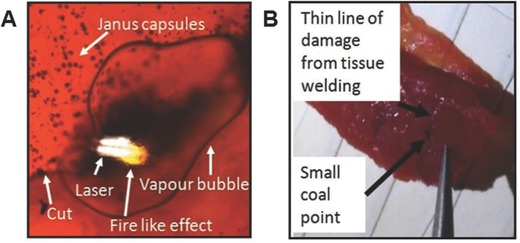
Tissue welding of meat A) showing fire like effects and vapor bubble due to strong local heat, image from Video S3 of the Supporting Information. As can be seen by the black line, more particles are in the cut than on the outside. The reason for more particles being on top side of cut is due to magnetic steering with magnet being on top side of sample while welding tissue; B) sealed meat sample, with small damage line from tissue welding, coal point from fire like effects, shows carbonized meat.

The IR temperature measurements on aforementioned meat samples and animals show that the achieved temperature increases significantly, whereby the integrated temperature is lower than 70 °C. Individual particles, especially aggregates achieve temperature higher than 100 °C, as fire like effects (starting point usually ≈250–365 °C)^29^, ^30^ on meat samples in **Figure** **3**A, as well as carbonized meat shown in Figure 3B prove. These fire like effects occurred when many particles agglomerated, causing local superheating. Such particle agglomerates were observed to occur randomly, whereby the local strong heating also caused water vapor bubbles as shown in Figure 3. In addition to fire like effect, we also observed thermal effects of blood. Blood agglomerated at 72 °C and red blood cells “melted” into a sort of film when agglomerated around a laser heated capsule. When a large number of blood cells agglomerated around the heated Janus capsule (or particle), a kind of “threshold” was overstepped, over which the blood cells suddenly turned into a film. This effect is probably caused by blood based NIR absorption, since blood shows a higher NIR absorption than water (Figure S5, Supporting Information). Examples of temperature and laser based effects on blood are shown in Figure S4 of the Supporting Information.

### 2.2 Laser Tissue Welding

**Figure 4:**
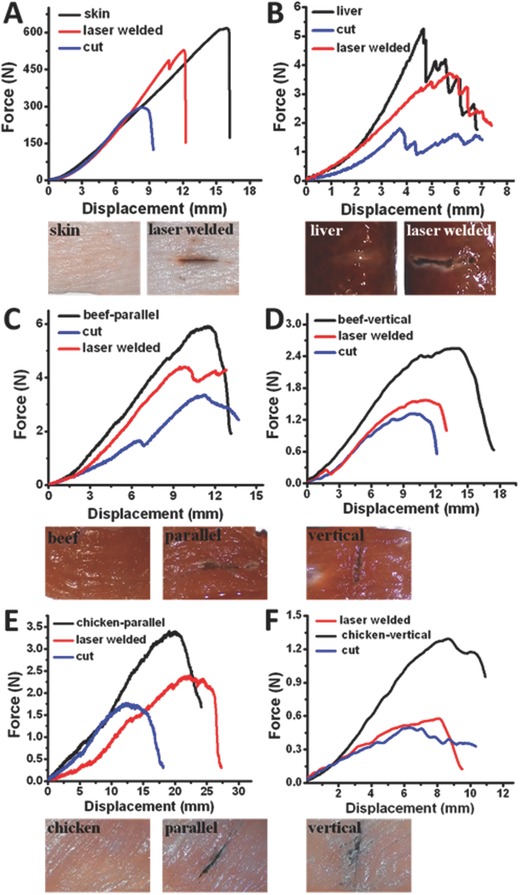
Proof of partial restoration of mechanical properties of A) skin, B) beef liver, C) beef meat parallel, D) beef meat vertical, E) chicken parallel, and F) chicken vertical alignment.

An interesting not resolved phenomenon is that the tissue welding causes a flow of particles into the laser area, which locally increases absorption, temperature and therefore overheating as Video S3 of the Supporting Information shows. The cause of this phenomenon is probably heat‐induced tissue shrinking. Mechanical measurements of bonded meat show a partial restoration of the meat's mechanical properties (see **Figure** **4**). The Janus capsule and laser based tissue welding was not able to fully restore the decreased mechanical properties of welded skin tissue. It was additionally found that the restoration capability is stronger for parallel than for vertical meat fiber alignment. This is because the collagen fibers condense in an amorphous manner, causing therefore the vertical meat fiber alignment to be recovered to a lower mechanical degree than the parallel meat fiber alignment. The cause of this is that the mechanical strength of polymers (to which collagen counts) is stronger along the fiber axis.^31^

These results are graphically displayed in Figure 4, with the pristine and welded meat samples being displayed in Figure S4C–F of the Supporting Information. The pig skin and the beef liver sample have no existing fibrous pre alignments. The fact that liver's mechanical properties can be restored with laser tissue welding offers the chance to utilize this kind of micromotor in the future for internal haemostatic treatments since livers as well as lung tissues can until now not be sutured.^4^, ^5^ The existing alternatives are blood coagulation factors, supporting thrombosis as well as nanoparticle gluing attempts for which one has to cut the patients open.^4^, ^5^

### 2.3 Healing Properties and Biocompatibility

**Figure 5:**
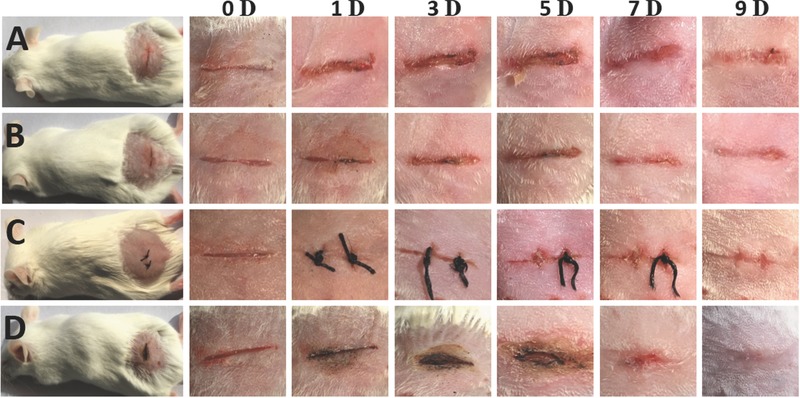
Examples of A) control sample (no treatment), B) laser tissue welding, C) medical suturing, D) nanoparticle glue. Scale bar: 5 mm. Only the nontreated wound heals worse than the treated wounds. Laser tissue welding based on autonomous movable dyes heals comparable to medical suturing or nanoparticle gluing.

Laser tissue welded mice exhibited similar thermal tissue damage like afore tested meat samples. The healing properties were however similar compared to nanoparticle gluing or medical suturing, despite the thermal damage. Only the nontreated wound healed significantly worse compared to treated wounds. We attribute these healing properties to the fact that the thermal damage was highly localized and microsized gold residues (as well as microsized carbons from thermal carbonized tissues) are not interfering with the healing process.^32^ In addition the wound was disinfected by temperature, which was not performed for the control samples (please note, no disinfection after wound treatment was performed, only prior to cutting mice were cleaned with 70% alcohol). Mice showed generally no infection signs due to sterile equipment. We note that medical suturing itself introduces additional injuries and tissue deformation, which was avoided in case of nanoparticle gluing and laser tissue welding. Healing example images of the mice are graphically displayed in **Figure** **5**. One can see that the healing was mostly finished for all treated wounds after ≈9 d. The nontreated wounds needed 2–3 d longer to heal.

**Figure 6:**
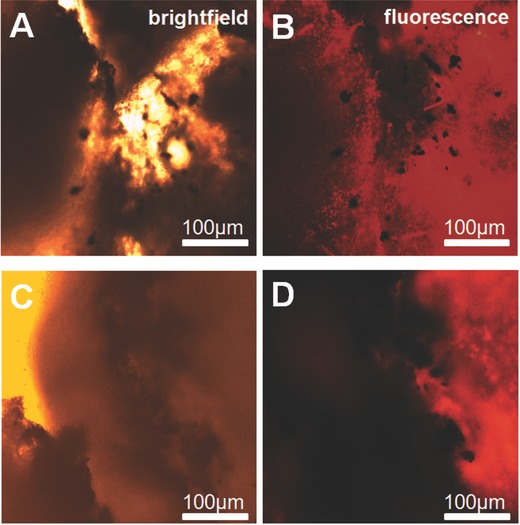
Histological slices stained with PI to determine the presence of dead cells after tissue welding, A,C) Bright field images, B,D) fluorescence images. (A) and (B) are investigated immediately after tissue welding, (C) and (D) after 9 d, whereby the cut was opened to better observe the particles. In (B) the dead cells are close to the incision and particles, which is not the case for (D).

The experiments suggest that a monolayer of nanoparticles perform similar healing effects as the welded tissue. As shown in **Figure** **6** the thermal damage is very small and localized and therefore the differences in healing are not observable from macroscopic point of view. The thermal damage was in range of 100–300 μm, which is ≈30 cells in diameter (assumed 10 μm long epithelial cells). A damage at this scale can be healed within a few days since not all cells within the said area are destroyed.

Histological analysis of pristine freshly welded samples using propidium iodide (PI) as a life‐dead stain a large amount of dead cells were found next to the welding site, which is due to highly localized heat. After 9 d the wound is mostly healed and the amount of dead cells next to the incision site is comparable with surrounding tissue, as observed in Figure 6. Also, the particles were found to stay close to the wound and did not affect the cells negatively.

**Figure 7:**
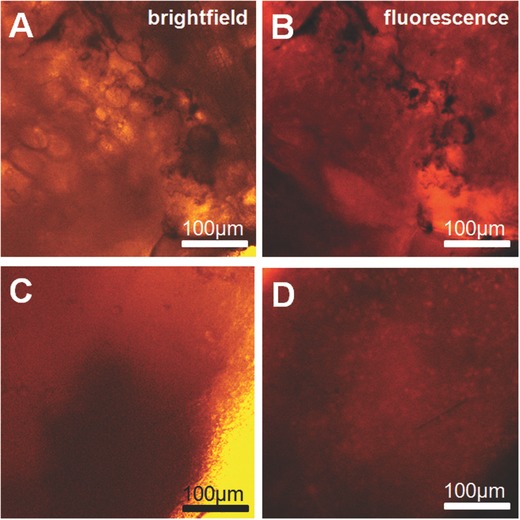
Histological slices of magnetite based nanoparticle gluing. A,C) are brightfield and B,D) PI fluorescence images for investigating dead cells (stained with PI). (A) and (B) are investigated immediately after gluing, (C) and (D) after 9 d. Magnetite nanoparticles are biocompatible, the gluing is not affecting surrounding cells and the particles are biodegradable.

This result is supported by a MTT test on the biocompatibility of the particles (see Figure S6, Supporting Information). During the test the biodegradation of Janus particles or capsules was not observed. Biodegradation is on the contrary quite obvious for magnetite nanoparticle based gluing. The magnetite particles show no adverse effects in PI based histological assays immediately after gluing and after 9 d the wound is nearly invisible and the particles are degraded as shown in **Figure** **7**.

**Figure 8:**
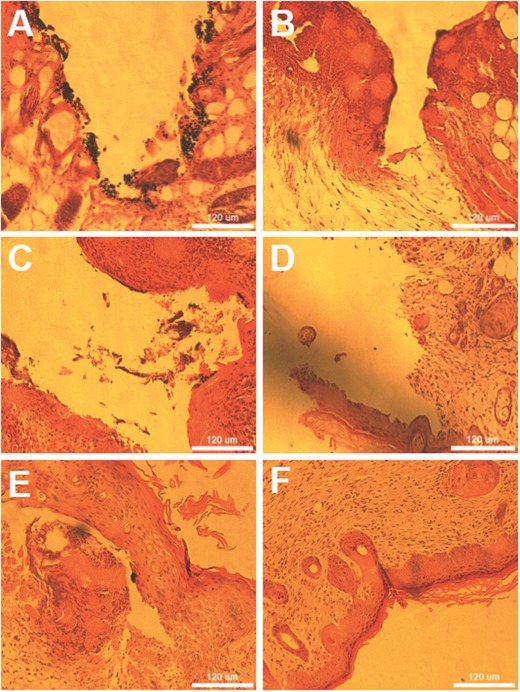
Hematoxylin and Eosin stained tissue slices A) 0 d, B) 1 d, C) 3 d, D) 5 d, E) 7 d, and F) 9 d after laser tissue welding. The particles agglomerate over time and are therefore removed by shear force during microtome slicing, gold removed by shear force can be seen as dark points. The tissue itself regenerates over time, despite initial thermal damage.

Using hematoxylin and eosin (H&E) stain one can determine the change of surrounding tissues due to laser assisted tissue welding. As shown in **Figure** **8**A, the Janus particles are clearly visible after the welding in a reopened cut. The tissue surrounding the cut is relatively inhomogeneous. After 1 d the cut still reopens during the staining procedure, whereby the tissue already renews itself and the particles are visible at the border (Figure 8B). With progressing time the cuts are more and more regenerated and stable, while the tissue continues to regenerate with a complete healing of ≈9 d. It is noted that the particles do not vanish over this period of time, but rather agglomerate and are removed by shear force during microtome slicing. This shear force causes the abrasion of gold on the particles which can be seen as black points in Figure 8D–F. H&E stained histological assay of different magnifications as well as H&E stained reference measurements of nanoparticle gluing can be observed in Figure S7 of the Supporting Information.

## 3 Conclusion

Janus micromotor particles driven by magnetic or thermal influences allow photothermal tissue welding. The healing properties of this photothermal tissue welding are comparable to medical suturing, or novel nanoparticle based tissue gluing. The ability to “guide” dyes in a wound allows for sealing bleeding wounds which would flush passive dyes as well as nanoparticles out. We additionally demonstrate that red blood cells can also be fused into a “film”, although its mechanical properties were not determinable in this study due to the presence of albumin, which is major tissue glue. Albumin presence was necessary in this study to facilitate a successful tissue welding. Histological investigations prove biocompatibility of the particles, as well as thermal damage due to tissue welding, whereby the damage due to tissue welding is restored within 9 d. The Janus particles remain within the tissue, in contrast to magnetite nanoparticle glue.

## 4 Experimental Section

*Methods and Materials*: The Janus capsules are based on a polyelectrolyte multilayer (PEM) shell. This PEM shell consists out of poly(styrenesulfonic acid) (PSS, *M*_w_ = 70 000) and poly(allylamine hyhrochloride) (PAH, *M*_w_ = 70 000), the anchoring layer^33^ on the silica template was polyethylenimeine (PEI, *M*_w_ = 750 000 g mol^−1^). All polyelectrolytes were purchased from Sigma, St. Louis, USA. The used ionic strength was 0.5 m NaCl (chemical reagent, Harbin, China), the used PE concentration 2g L^−1^. The utilized silica sphere templates were 1, 5, and 20 μm diameter spherical particles acquired from Baseline, Tianjin, China. The assembly on top of the particles was performed according to Donath et al.^34^ Briefly silica particles were inserted into a vial, then PEI solution was added and the particles were shaken for 10 min whereby the particles were subsequently centrifuged and the PE solution was decanted away. Three washing steps in water (ultrapure water, resistance >18.2 MΩ cm, Elga labwater, Beijing, China) with 30 s immersion time were used to remove nonadsorbed PE. In a following step oppositely charged PE solution was used to complete the first bilayer, followed by again three washing steps and PAH solution. PSS and PAH solutions were alternatively deposited until five bilayers were assembled.

After the final washing step, the PEM coated particles were deposited as a monolayer onto a glass slide and ≈100 nm of gold was sputtered onto the particles, creating a Janus‐like structure. Afterward the particles were removed from the glass and the core of the particles was dissolved in 0.3 m HF (caution, HF is toxic and can penetrate the skin). After three washing steps, the Janus capsules were obtained. It is noted, that prior to Janus structure creation 2–3 bilayers of superparamagnetic Janus particles (prepared according to Lee et al.^35^) and PAH were assembled onto the particles to enable magnetic guidance.

*Characterization*: The particles were characterized via scanning electron microscopy (SEM) (S‐5200 Tokyo, Japan). The UV–vis‐NIR absorption spectra were acquired by a Hitachi U‐4100 UV–vis‐NIR spectrometer (Hitachi, Tokyo, Japan). A Leica TCS SP5 II confocal laser scanning microscope (CLSM) (Leica, Heidelberg, Germany) was used to obtain the confocal images. The excitation wavelength for FITC was 488 nm.

*Laser Tissue Welding, Thermophoresis, and Temperature Determination*: A modified inverted microscope (Olympus IX 71, Tokyo, Japan) was used to acquire optical images of the particles, as well as to perform laser based tissue welding (many particles) and temperature measurements on single particle level. The camera was a Qcam (Qimaging B series, Surrey, Canada). The image acquisition software was Qimaging Pro 7.0 with an exposure time of 100 mS. The fastest image acquisition rate was 1 image per second this is why Videos S1–S3 of the Supporting Information look rugged. The laser for heating the particles was a multimode laser (MW IR 808 2000 mW) with wavelength of 808 nm, and was housed on an ATR‐1800 manual laser power control unit (both FS‐Optics, Shanghai, China). The optical trapping experiments for temperature measurements used for comparison were performed with an 808 nm TEM00 mode laser with maximum power of 80 mW (Shanghai Laser & Optics Century Co., Shanghai, China). Temperature measurements were done by relating the temperature to diffusion utilizing a method originally developed for optical tweezers^36^, ^37^, ^38^ The basic underlining equation of diffusion in relation to temperature was is the Stokes–Einstein–Sutherland equation.^39^, ^40^ Position measurements for power spectra were done, by laser deflection of the particle, which causes the laser to illuminate different areas of a quadrupole diode with different intensities, depending on the particle position. The used quadrupole diode was a PDS‐196‐LC (Octron, Shanghai, China) whereby the data were collected with a NI‐6008 (National Instruments, Austin, TX, USA) read out card. For optical trapping of large particles 20 × APO illumination objective with a 10 × APO laser collection objective (both objectives Olympus, Tokyo, Japan) was used. For 1 and 5 μm particles a 40 × APO illumination objective and a 20 × APO collection objective was used. The XYZ‐alignment of the collection objective was achieved using a home‐made alignment stage comprising 3 TSM‐25 micropositioning tables (Zolix, Beijing, China). Contrary to the temperature determination most wound sealing was performed using a 4 × plano objective (Olympus, Tokyo, Japan). Dynamic viscosity data for water was obtained from ref. 41 which contains a reliable online library of technical data. The difference in particle diffusion rate was compared with pure silicon dioxide, Janus‐PEM particles (core was not dissolved for optical trapping and laser deflection reasons) as well as simulated particle diffusion rate of different temperatures, by employing the Stokes–Einstein–Sutherland equation.^39^, ^40^ The used approach introduces an error due to different light scattering abilities between gold nanoshell and pure silicon particles. This error was bypassed by normalizing the laser power of the Janus particles with the one of pristine particles for laser powers when optical trapping the particles starts. For details see Section 2.

*Mechanical Measurements and Used Samples*: The used meat samples comprised out of beef muscle tissue, chicken muscle tissue, beef liver, and pig skin which were acquired from a local butchery in Harbin, P. R. China. The meat samples were cut with a scalpel into sizes of 2 × 4 cm^2^ and a thickness of 5 mm. A tensiometer (5900 Dual Column Floor Model Testing System, Grove City, USA) was used to measure the mechanical properties of pristine, lased, and laser welded meat. The introduced cut was 3 × 10 mm^2^. The elongation speed of the tensionmeter was 10 mm min^−1^. It is noted, that fibrous tissue such as muscle meat exhibits different mechanical strength depending on orientation and was therefore tested in vertical and parallel orientation to the fibers. For this reason, the meat was also cut parallel and vertical toward the muscle fibers. Animal experiments were performed using female BALB/C white mice, supplied by the life science department of HIT. The animal experiments were performed according to the ethical guidelines of the life science department. During procedure the mice were anesthetized by injecting 0.1 mL of 2.5% sodium pentobarbital. For histological investigations mice were euthanatized by injecting a lethal dose of sodium pentobarbital.^42^ Figure S8 of the Supporting Information shows the magnetic field arrangement during particle guidance. During magnetic particle guidance the magnetic field was arranged 90° in *θ* compared to cutting direction to guide the particles into the cut. The field was moved ±180° in Φ angle as needed, depending if particle sedimentation or guidance was needed.

We compared laser based sealing properties also with the nanoparticle tissue gluing method of Leibler and co‐workers^4^, ^5^ to evaluate the healing properties. The used particles for nanoparticle gluing experiments were superparamagnetic magnetide nanoparticles, also used for steering our capsules and particles. The choice for this kind of particles is their biodegradability and biocompatibility.

For laser tissue welding experiments the mice were shaved and a 5 mm long and 2–3 mm deep cut was made on the back. Then particles and 1 drop of mouse blood (from same mouse) were inserted and the wound was closed with laser. Comparison with medical suturing and no aid to the wound were made. To compare and visualize photothermal effects optically we additionally used an IR camera (MR 170, FLIR, Wilsonville, Oregon, USA) to observe heating on animals, meat and in buffer as well as on dry glass slides.

## Supporting information

As a service to our authors and readers, this journal provides supporting information supplied by the authors. Such materials are peer reviewed and may be re‐organized for online delivery, but are not copy‐edited or typeset. Technical support issues arising from supporting information (other than missing files) should be addressed to the authors.

Supplementary

Supplementary

Supplementary

Supplementary
