# Supplementary material for: Guidable Thermophoretic Janus Micromotors Containing Gold Nanocolorifiers for Infrared Laser Assisted Tissue Welding
Source: Adv Sci (Weinh). 2016 Sep 1;3(12):1600206. doi: 10.1002/advs.201600206 (PMC5157175; doi:10.1002/advs.201600206)
Supplement: Supplementary file 1 — Supplementary [file ADVS-3-0-s001.pdf]

Copyright WILEY-VCH Verlag GmbH & Co. KGaA, 69469 Weinheim, Germany, 2016.

Supporting Information

for *Adv. Sci.,* DOI: 10.1002/advs.201600206

Guidable Thermophoretic Janus Micromotors Containing Gold Nanocolorifiers for Infrared Laser Assisted Tissue Welding

Wenping He, Johannes Frueh, Narisu Hu, Liping Liu, Meiyu Gai, and Qiang He**

Copyright WILEY-VCH Verlag GmbH & Co. KGaA, 69469 Weinheim, Germany, 2013.

Supporting Information

Guidable Thermophoretic Janus Micromotors Containing Gold Nanocolorifiers for Infrared Laser Assisted Tissue Welding

Wenping He[1], *Johannes Frueh*[*1], *Narisu Hu*[1], *Liping Liu*[2], *Meiyu Gai*[1,3], *Qiang He*[*1]

[1]Key Laboratory of Microsystems and Microstructures Manufacturing, Ministry of Education, Micro/Nano Technology Research Centre, Harbin Institute of Technology, Yikuang Street 2, Harbin 150080, P. R. China, email: Johannes.Frueh@hit.edu.cn, Qianghe@hit.edu.cn
[2]Mental Health Centre, 1st Affiliated Hospital of Harbin Medical University, Harbin 150001, PR China
[3]Queen Mary University of London, Mile End, Eng, 215, London E1 4NS, United Kingdom

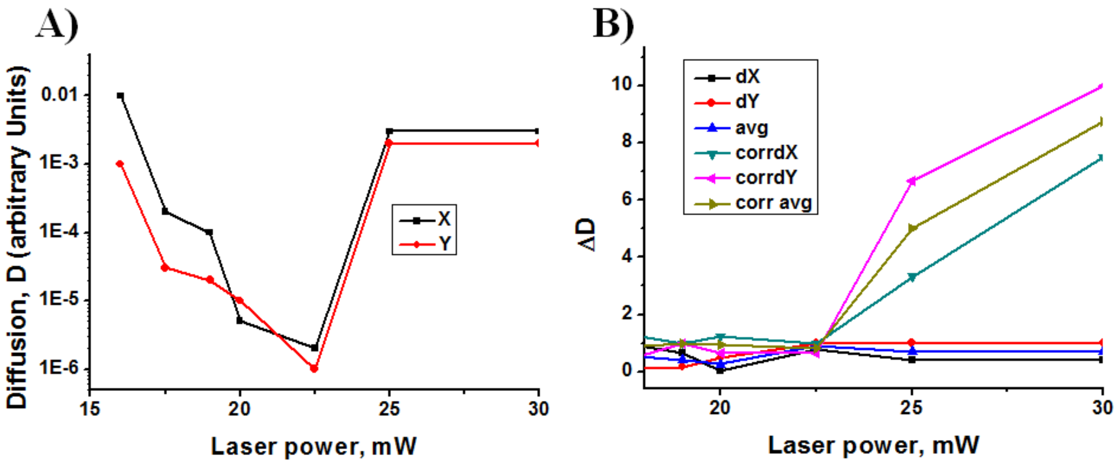

Figure S1. Comparison between (A) diffusion of particle versus laser power and (B) relative and trapping corrected relative diffusion (corr) of a particle at different laser powers in X and Y dimention. Average of X and Y is labelled avg in (B).

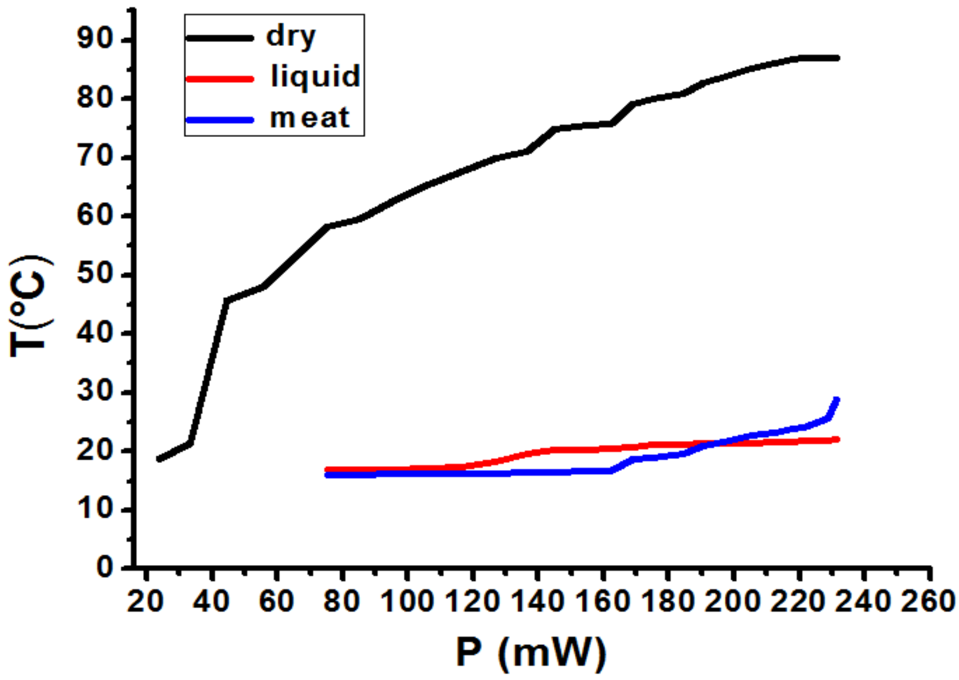

Figure S2. Dependence of detected temperature with laser power (P) using a standard heat camera and dried Janus particles on glass slide, Janus particles in liquid and Janus particles on meat. Images of these measurements are shown in Supporting Information Figure S3.

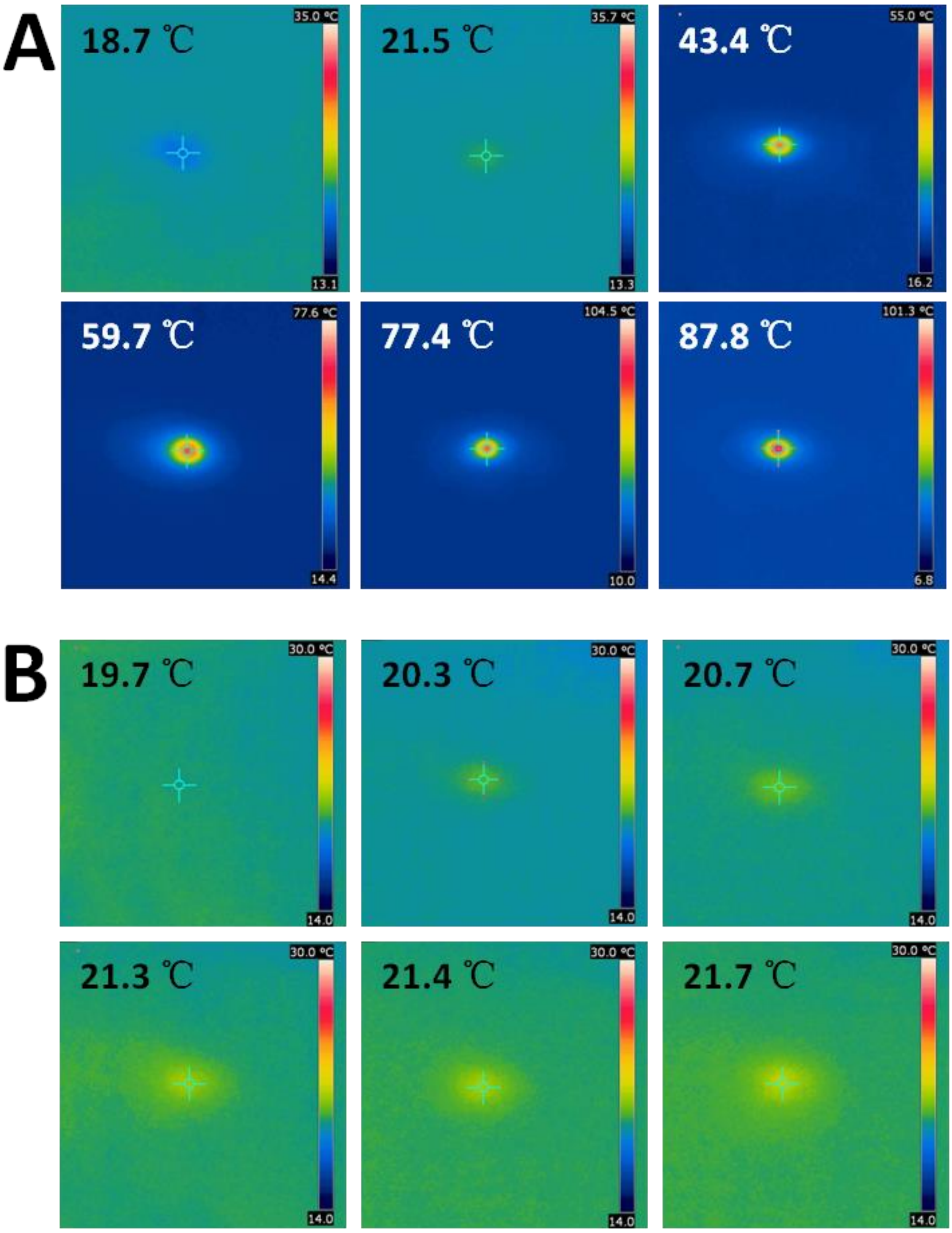
A
18.7 ℃
35.0 ºC
13.1
21.5 ℃
35.7 ºC
13.3
43.4 ℃
55.0 ºC
16.2
59.7 ℃
77.6 ºC
14.4
77.4 ℃
104.5 ºC
10.0
87.8 ℃
101.3 ºC
6.8
B
19.7 ℃
20.3 ℃
20.7 ℃
21.3 ℃
21.4 ℃
21.7 ℃
30.0 ºC
14.0

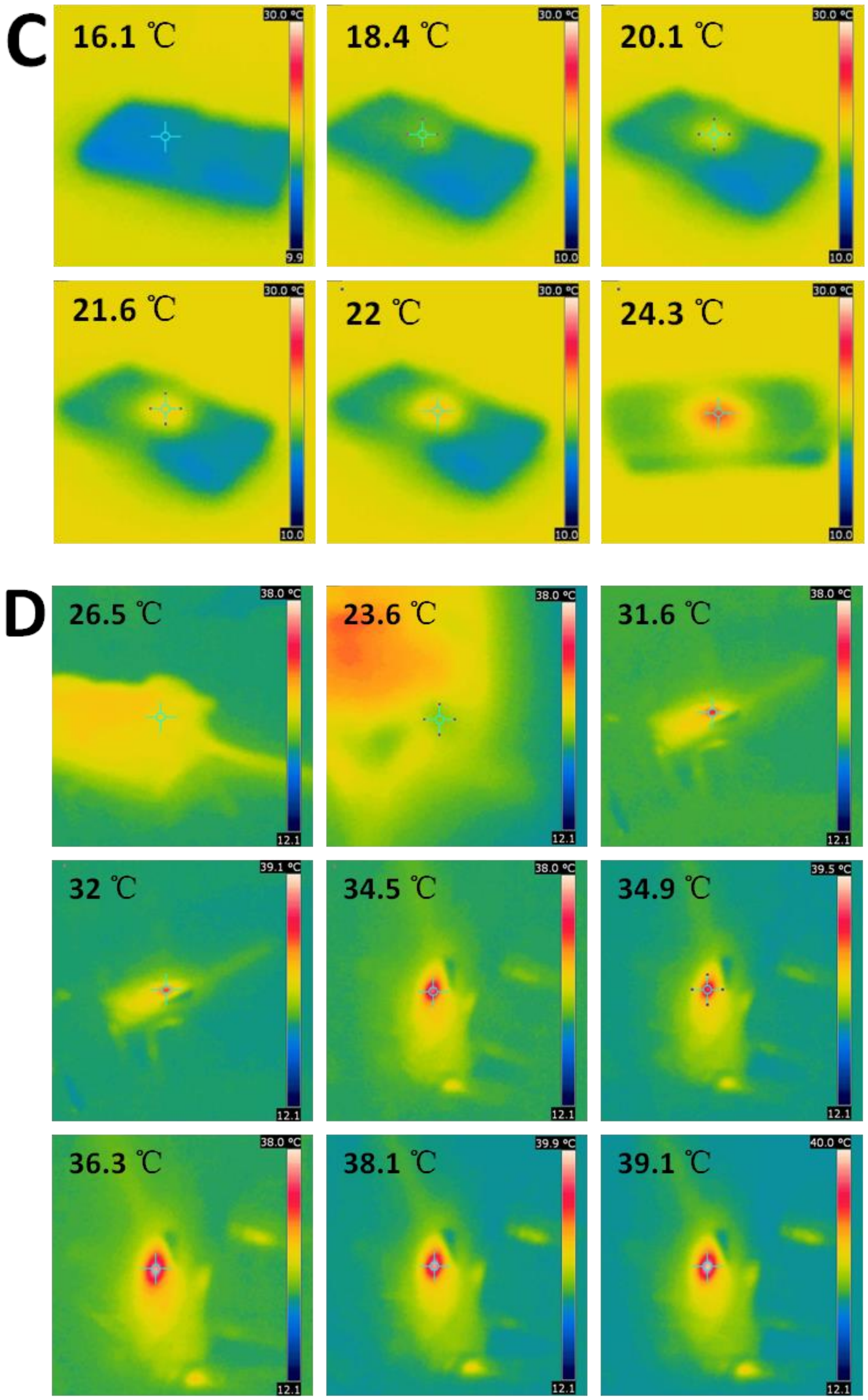

Figure S3. Laser based heating of Janus micromotors with intensities displayed in Figure S2. (A) dry state; (B) PBS solution; (C) meat; (D) mouse. Color shifts due to re-normalization of the camera depending on hottest point.

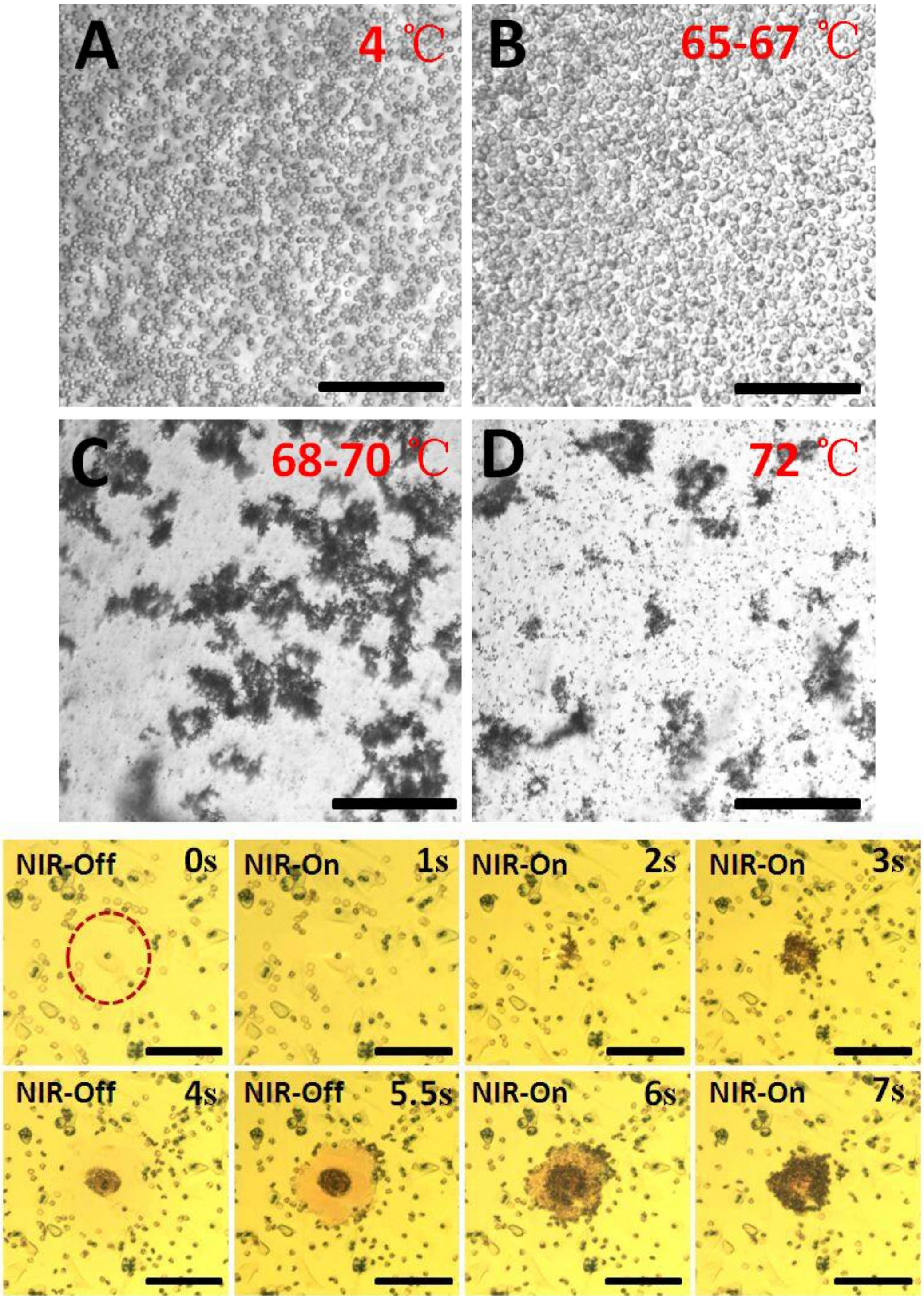

Figure S4. Thermal behavior of blood and blood cells. (A-B) From ambient until 65 °C blood cells stay dispersed but agglomerate at 68-70°C (C-D). Scale bar = 200 µm. Using a Janus particle trapped on a surface, one can create a local hotspot using laser irradiation, whereby the fluid flow attracts red blood cells which themselves adsorb light (E). If a certain threshold concentration is reached, the red blood cells melt into a film (5-6s).

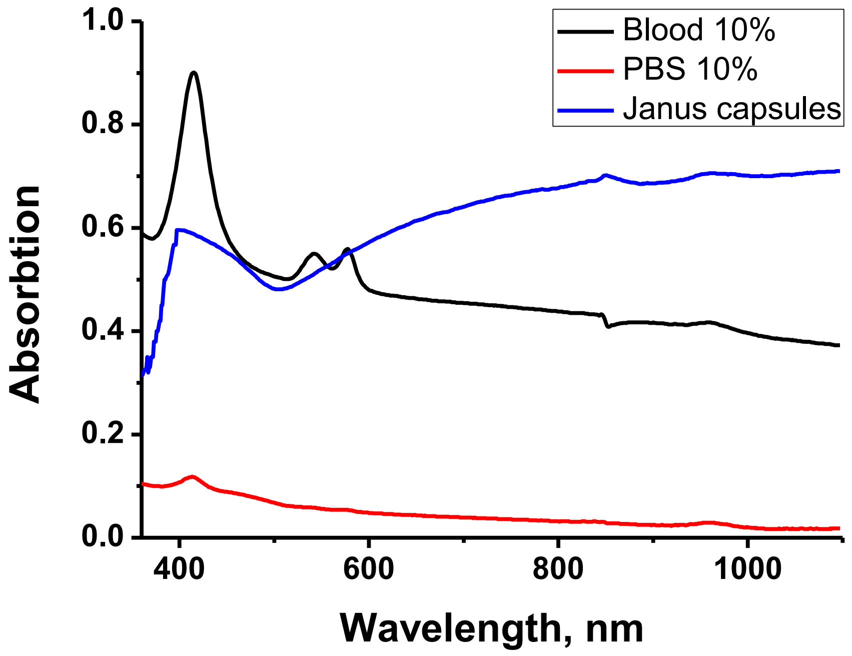

Figure S5. Absorption of blood, PBS and Janus capsules. Blood cells absorb also at NIR, however less than Janus capsules.

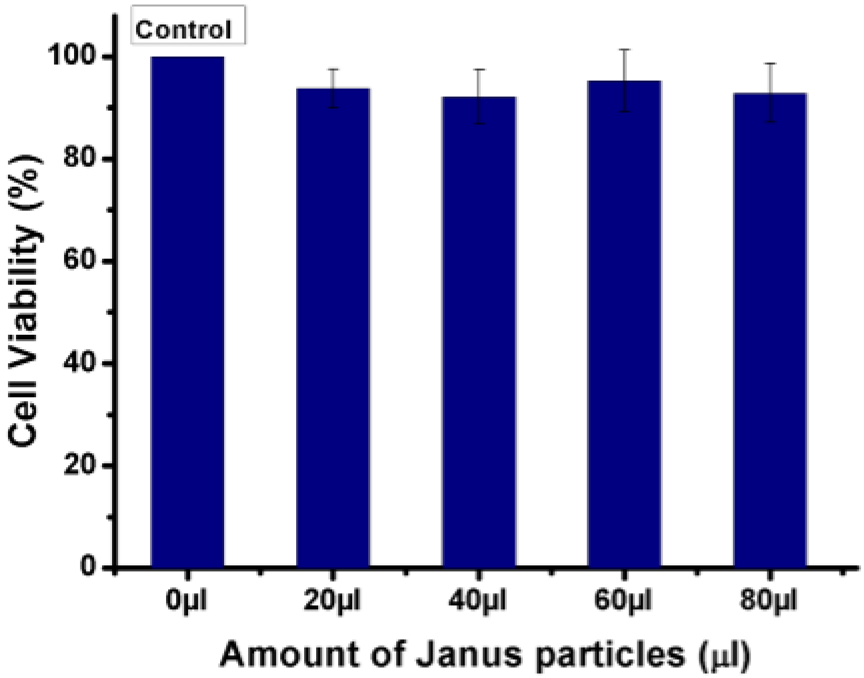

Figure S6. MTT test proving non-cytotoxicity of the Janus particles.

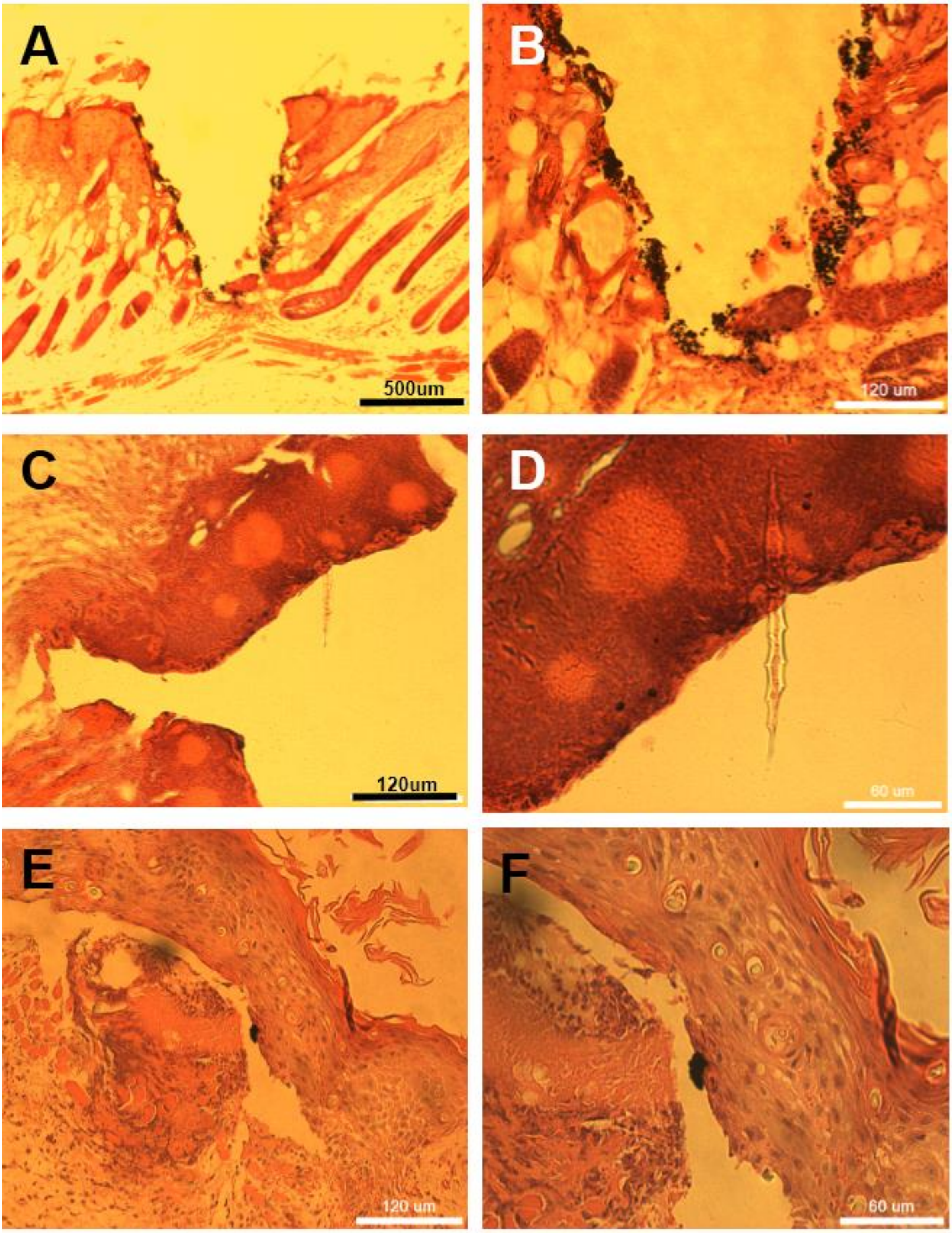
A
500um
B
120 um
C
120um
D
60 um
E
120 um
F
60 um

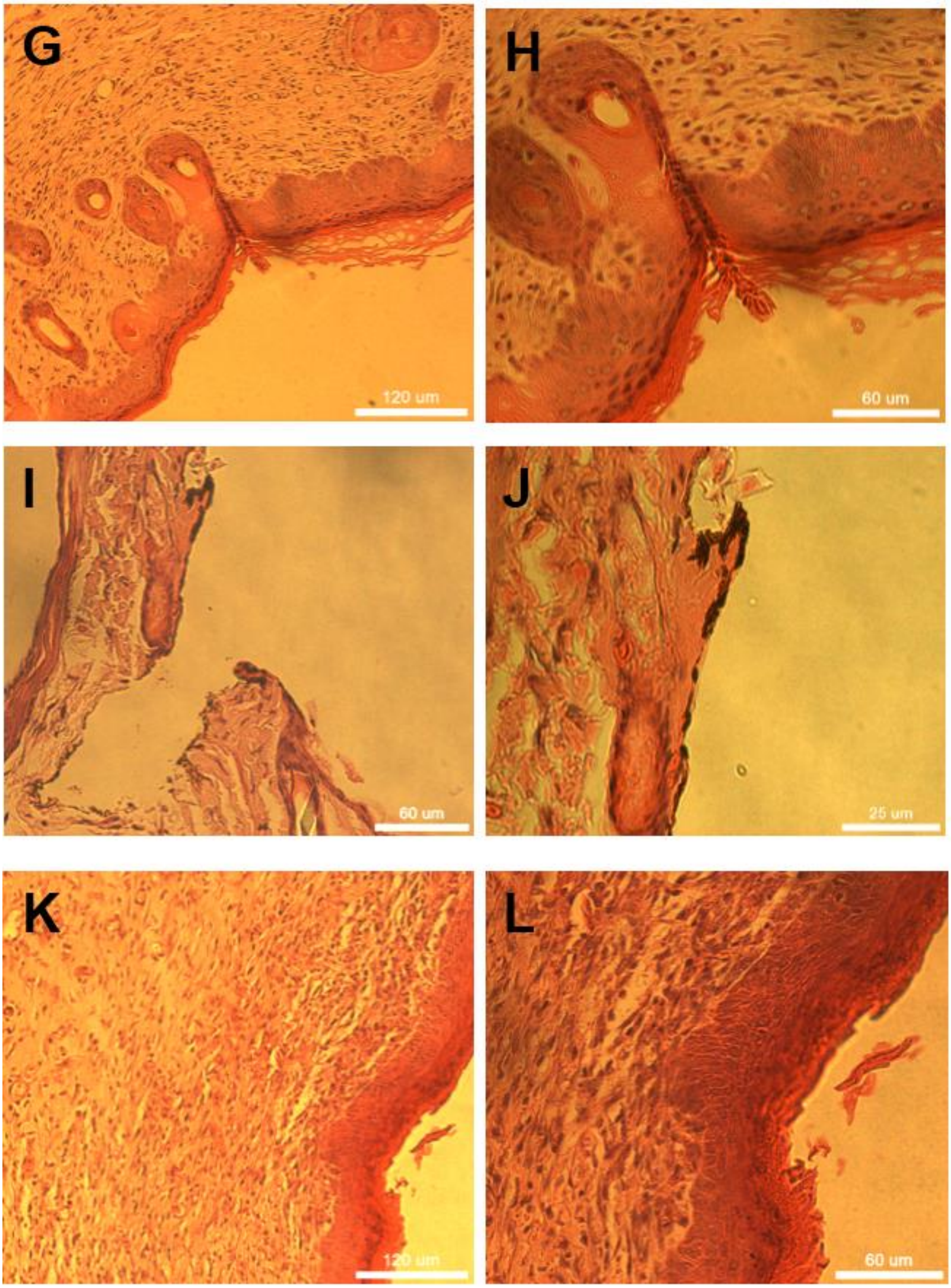

Figure S7. Histological slices stained with hematoxylin and eosin of laser tissue welded tissue after 0 days at 4 × (A) and 10 × (B) zoom, 1 day at 10 × (C) and 20 × (D) zoom, 7 days at 10 × (E) and 20 × (F) zoom, 9 days at 10 × (G) and 20 × (H). Fresh nanoparticle glued tissue at 10 × (I) and 20 × (H) zoom and 9 day old nanoparticle glued tissue at 10 × (K) and

20 × (L) zoom. The healing after 9 days of laser tissue welded and nanoparticle glued tissue is macroscopically but not microscopically comparable.

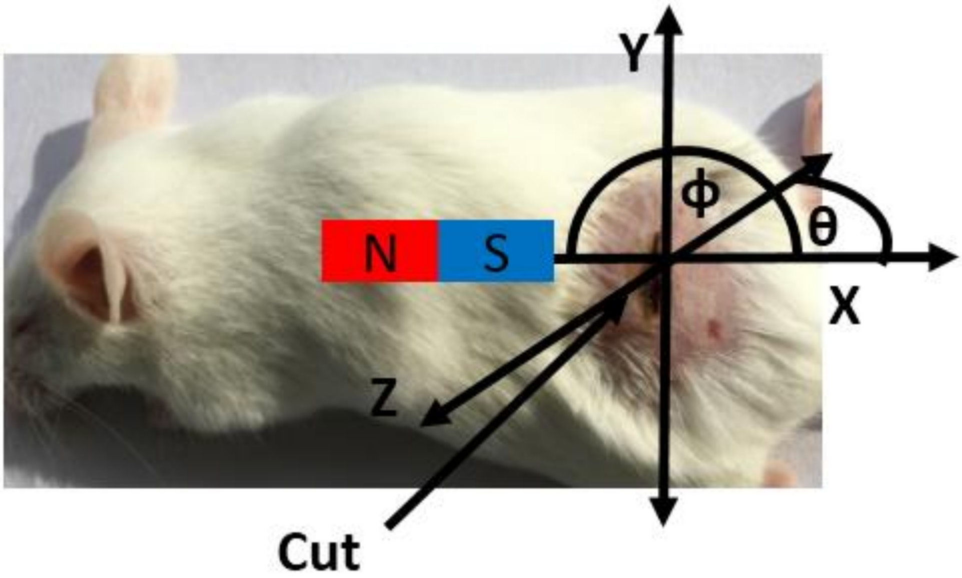

Figure S8. Arrangement of magnetic field during magnetic particle guidance, the orientation of the angles θ in X-Z and ϕ in XY space can be observed.
